# Antibody-drug conjugates for lymphoma patients: preclinical and clinical evidences

**DOI:** 10.37349/etat.2022.00112

**Published:** 2022-12-26

**Authors:** Marilia Barreca, Noémie Lang, Chiara Tarantelli, Filippo Spriano, Paola Barraja, Francesco Bertoni

**Affiliations:** 1 Department of Biological, Chemical and Pharmaceutical Sciences and Technologies (STEBICEF), University of Palermo, 90123 Palermo, Italy; 2 Division of Oncology, Department of Oncology, Faculty of Medicine, Geneva University Hospitals, 1205 Geneva, Switzerland; 3 Institute of Oncology Research, Faculty of Biomedical Sciences, USI, 6500 Bellinzona, Switzerland; 4 Oncology Institute of Southern Switzerland, Ente Ospedaliero Cantonale, 6500 Bellinzona, Switzerland; Istituto Nazionale Tumori “Fondazione Pascale” Via Mariano Semmola, Italy

**Keywords:** Antibody-drug conjugate, cytotoxic payload, monoclonal antibody, linkers, lymphoma

## Abstract

Antibody-drug conjugates (ADCs) are a recent, revolutionary approach for malignancies treatment, designed to provide superior efficacy and specific targeting of tumor cells, compared to systemic cytotoxic chemotherapy. Their structure combines highly potent anti-cancer drugs (payloads or warheads) and monoclonal antibodies (Abs), specific for a tumor-associated antigen, via a chemical linker. Because the sensitive targeting capabilities of monoclonal Abs allow the direct delivery of cytotoxic payloads to tumor cells, these agents leave healthy cells unharmed, reducing toxicity. Different ADCs have been approved by the US Food and Drug Administration (FDA) and the European Medicines Agency (EMA) for the treatment of a wide range of malignant conditions, both as monotherapy and in combination with chemotherapy, including for lymphoma patients. Over 100 ADCs are under preclinical and clinical investigation worldwide. This paper it provides an overview of approved and promising ADCs in clinical development for the treatment of lymphoma. Each component of the ADC design, their mechanism of action, and the highlights of their clinical development progress are discussed.

## Introduction

Antibody-drug conjugates (ADCs) are currently among the most appealing anti-cancer therapeutic tools, and their development is expected to further increase [1–6]. ADCs have realized the concept of the “magic bullets”, i.e. chemicals specifically targeting microbes or cancer cells, developed by Ehrlich at the beginning of the 20th century [7, 8], fully exploiting the discovery of the hybridoma technology by Köhler and Milstein [9] in 1975 to produce mouse monoclonal antibodies (Abs).

The first generation of ADCs mostly failed in showing clinical benefit due to the use of murine Abs, recognized as non-self by the human immune system [10]. The generation of human anti-mouse antibodies (HAMAs) accelerated the clearance of ADCs, reducing their pharmacokinetics and the delivery of the drug in tumor site [10–12]. In addition, the choice of antigens highly expressed in normal cells caused serious side effects, linkers were unstable in human blood flow (inducing ADCs’ short half-life) and only 1–2% of the dose reached cancer cells, hence the therapeutic efficacy was not satisfactory [3]. Also, thanks to the pioneering work of Winter on “humanized” mouse monoclonal Ab [13], more rational designs led to the first ADC approval by Food and Drug Administration (FDA) in 2000: gemtuzumab ozogamicin for patients with CD33-positive acute myeloid leukemia. To date, eleven ADCs have received market approval from the FDA (Table 1). Among them, three have been approved for the treatment of lymphomas: brentuximab vedotin, polatuzumab vedotin, and loncastuximab tesirine. In this review, after providing a general overview of ADCs, we will focus on their application for patients with lymphomas, since several compounds are currently under evaluation both in preclinical and clinical stages for lymphoma patients (Table 2).

**Table 1.** ADCs approved by FDA sorted based on the year of their first approval

**ADC**	**Target**	**Disease with FDA approval**	**Approval year**
Gemtuzumab ozogamicin	CD33	Acute myeloid leukemia	2000^*^, 2017
Brentuximab vedotin	CD30	ALCL, CTCL, HL, MF, PTCL	2011^*^, 2018
Trastuzumab emtansine	HER2	Breast cancer	2013
Inotuzumab ozogamicin	CD22	Lymphoblastic leukemia-lymphoma	2017
Moxetumomab pasudotox	CD22	HCL	2018
Polatuzumab vedotin	CD79B	DLBCL	2019
Enfortumab vedotin	Nectin-4	Urogenital cancer	2019
Trastuzumab deruxtecan	HER2	Breast cancer, gastric cancer	2019
Sacituzumab govitecan	Trop-2	Breast cancer, urogenital cancer	2020
Belantamab mafodotin	BCMA	Multiple myeloma	2020
Loncastuximab tesirine	CD19	DLBCL	2021

* approved as monotherapy; ALCL: anaplastic large cell lymphoma; BCMA: B cell maturation antigen; CTCL: cutaneous T cell lymphoma; DLBCL: diffuse large B cell lymphoma; HCL: hairy cell leukemia; HER2: human epidermal growth factor receptor 2; HL: Hodgkin’s lymphoma; MF: mycosis fungoides; PTCL: peripheral T cell lymphoma

**Table 2.** List of ADCs that have entered the clinical evaluation for lymphoma patients, sorted by their target and by their official name, if assigned, or by their common/alternative name

**Target**	**Alias**	**Development codes**	**Linker**	**Payload**	**Payload target**	Clinical stage^*^	Orphan drug status (if any)^*^	Development stage^*^	**Key AEs**
CD19	Coltuximab ravtansine [111, 188]	SAR3419, huB4-DM	SPDB (C)	DM4	Microtubules	2	-	No on-going trials	IRR, ocular toxicity^±^, gastrointestinal toxicity^†^, peripheral neuropathy
	Denintuzumab mafodotin [112, 189]	SGN-CD19A, SGN-19A, hBU12-491	Maleimidocaproyl (NC)	MMAF	Microtubules	2	-	No on-going trials	Ocular toxicity^±^
	Loncastuximab tesirine [113, 115, 121]	ADCT-402, RB4v1.2- SG-3249	Val-Ala (C)	PBD (SG3199)	DNA	Appr.	MCL, DLBCL	On-going trials	IRR, thrombocytopenia, neutropenia, cutaneous toxicity, edema/effusion
CD20	-	MT-3724 [190–193]	Ab directly fused to toxin	Shiga-like toxin-I A1	Ribosomes	2	-	No on-going n.a. trials	
CD22	Inotuzumab ozogamicin [133, 194, 195]	CMC-544, PF-5208773, WAY-207294	4-(4-acetylphenoxy) butanoic acid (C)	Calicheamicin	DNA	2	Precursor cell lymphoblastic leukemia-lymphoma	On-going trials	IRR^‡^, neutropenia, febrile, thrombocytopenia, anemia, gastrointestinal toxicity^†^, hepatotoxicity^§^, pancreatitis/ lipase elevation, veno-occlusive disease/sinusoidal obstruction syndrome, hemorrhagic events, tumor lysis syndrome
	Pinatuzumab vedotin [132, 149]	DCDT2980S, RG-7593, ACD22- VCMMAE, FCU-2703, RG-7593, RO-5541072	Val-Cit (C)	MMAE	Microtubules	2	-	No on-going trials	IRR^‡^, neutropenia, peripheral neuropathy, gastrointestinal toxicity^†^, hyperglycemia
	Moxetumomab pasudotox [136, 196]	RFB4[GTHW] , (dsFv)-PE38, HA22, CAT- 8015	-	*Pseudomonas* exotoxin A (PE38)	Protein synthesis	Appr.	HCL	On-going trials	IRR, gastrointestinal^†^, edema, anemia, hypophosphatemia, headaches, hemolytic uremic syndrome, capillary leak syndrome
	Epratuzumab- cys-tesirine [135]	ADCT-602, Epratuzumab-cys-SG3249, hLL2-cys-PBD, hLL2-cys-SG3249	Val-Ala (C)	PBD (SG3199)	DNA	2	-	On-going trials	n.a.
	-	TRPH 222, CAT-02-106 [134, 140]	SMARTag (NC)	Maytansinoid	Microtubules	1	-	On-going trials	n.a.
CD25	Camidanlumab tesirine [155, 157, 158, 197, 198]	ADCT-301	Val-Ala (C)	PBD (SG3199)	DNA	2	-	On-going trials	IRR, gastrointestinal^†^, anemia, hepatotoxicity, skin, edema, headache, immune system disorder and multiple cranial nerve palsy
CD30	Brentuximab vedotin [66, 68, 76, 199–202]	SGN-35, cAC10- vcMMAE	Val-Cit (C)	MMAE	Microtubules	Appr.	HL, ALCL, PTCL, CTCL	On-going trials	IRR^‡^, gastrointestinal toxicity^†^, neutropenia, thrombocytopenia, peripheral neuropathy, hyperglycemia, pancreatitis/ lipase elevation, pneumonitis, hepatotoxicity^§^, JCV-induced progressive multifocal leukoencephalopathy
	-	F0002-ADC, anti-CD30- MCC-DM1 [94]	SMCC (NC)	DM1	Microtubules	1	-	On-going trials	n.a.
CD37	Naratuximab emtansine [161, 162, 166]	Debio 1562, IMGN529	SPDB (C)	DM4	Microtubules	2	DLBCL	No on-going trials	IRR, gastrointestinal toxicity^†^, febrile neutropenia, thrombocytopenia, tumor lysis syndrome
	-	AGS67E [203, 204]	Maleimidocaproyl- Val-Cit-PABC (C)	MMAE	Microtubules	1	-	No on-going trials	n.a.
CD70	-	MDX-1203, BMS936561 [205, 206]	Val-Cit (C)	Duocarmycin (MED-2460)	DNA	1	-	No on-going trials	n.a.
	Vorsetuzumab mafodotin [207]	SGN-75, h1F6- mcMMAF	Val-Cit (C)	MMAE	Microtubules	1	-	No on-going trials	n.a.
	-	SGN-CD70A, SGN 70A [169]	Maleimidocaproyl 1 (NC)	MMAF	Microtubules	1	-	No on-going trials	n.a.
CD71	-	CX-2029, ABBV-2029 [20, 172]	Val-Cit (C)	MMAE	Microtubules	2	-	On-going trials	n.a.
CD79B	Polatuzumab vedotin [28, 96, 98, 149]	DCDS4501A, RG7596, anti-CD79B- VC-MMAE, RO-5541077	Val-Cit (C)	MMAE	Microtubules	Appr.	DLBCL	On-going trials	IRR^‡^, gastrointestinal toxicity^†^, febrile neutropenia, anemia, thrombocytopenia, peripheral neuropathy, edema
	Iladatuzumab vedotin [107]	DCDS0780A, RO7032005	Maleimidocaproyl- Val-Cit-PABC (C)	MMAE	Microtubules	1	-	No on-going trials	n.a.
CD205	-	MEN1309, OBT076 [174]	*N*-succinimidyl- 4-(2-pyridyldithio) butanoate (C)	DM4	Microtubules	1	-	On-going trials	n.a.
ROR1	Zilovertamab vedotin [177–179, 182]	VelosBio101, MK 2140, VLS-101	Maleimidocaproyl-Val-Cit-*para-*aminobenzoate (C)	MMAE	Microtubules	2	MCL	On-going trials	n.a.
	-	NBE-002 [180]	Sortase A-mediated SMAC-technology (NC)	PNU-159682	DNA	1	-	On-going trials	n.a.

* based on http://adisinsight.springer.com and/or https://clinicaltrials.gov accessed in June 2022; -: none; ±: including corneal, extra corneal, and lacrimal disorders;

†: including nausea, vomiting, and diarrhea;

‡: including headache, rash, back pain, vomiting, chills, nausea, dyspnea, pruritus, cough, and anaphylaxis;

§: including liver enzyme and/or bilirubin elevation; AEs: adverse events; Ala: alanine; Alias: international nonproprietary name; Appr.: approved; C: cleavable; Cit: citrulline; DM1: maytansinoid, mertansine; DM4: maytansinoid, ravtansine; IRR: infusion-related reaction; MMAF: monomethyl auristatin F; MCL: mantle cell lymphoma; n.a.: data not available; NC: not cleavable; PABC: *p*-amino benzyloxycarbonyl; PBD: pyrrolobenzodiazepine; ROR1: receptor tyrosine kinase-like orphan receptor 1; SMCC: succinimidyl *trans*-4-(maleimidylmethyl)cyclohexane-1-carboxylate; SPDB: *N*-succinimidyl 4-(2-pyridyldithio)butyrate; Val: valine

## Composition and design of ADCs

In this section, we will describe the different properties of components of an ADC construct: target, Ab, linker, and payload.

### Target

ADCs are highly complex biopharmaceutical drugs composed of three parts: the Ab, the cytotoxic agent, and the linker that joins the first two components [6, 10, 14–19]. The development of a successful ADC starts with the identification of an appropriate target antigen for the Ab component. Ideally, the target should be expressed only or preferentially on tumor cells with negligible levels in healthy tissues, to minimize off-target efficacy. The development of ADC prodrugs is aimed to improve the specificity of activating the actual compounds only at the tumor site exploiting, for example, the presence of proteases [16, 20]. The selected antigen should also have a consistent expression on the targeted cell population surface to be available to the monoclonal Ab [15, 21]. It is important to mention the so-called ‘bystander effect’, in which payloads can diffuse into surrounding cells and still exert their cytotoxic effects, even on cells expressing lower or no target [22].

In general, albeit this might not fully apply to the lymphoma field, antigens that are abundant in blood circulation should also be avoided to prevent sequestration and/or degradation of the ADCs before reaching the tumor site.

Requirement in target antigen selection is also the efficacy in trafficking ADC into tumor cells. The internalization process, also known as receptor-mediated endocytosis, is crucial to ensure ADC transport into intracellular compartments where the payload is released through proteolytic degradation of the Ab moiety and/or cleavage of the linker.

Besides determining a lack of efficacy, inadequate and/or inefficient ADC internalization increases the risk of toxicity due to the drug release in the extracellular space. Other factors that influence the rate of internalization are the epitope selectivity, since different Ab-epitopes have different interaction kinetics, and the binding affinity of the ADC to the antigen. The binding between the epitope (antigenic determinant on the cell) and the paratope (antigen-combining site on the Ab) depends on noncovalent bonds such as hydrogen bonds, hydrophobic interactions, electrostatic bonds, or Van der Waals forces [23].

Different mechanisms for target-independent uptake, or unspecific uptake, may also contribute to ADC internalization. Pinocytosis, also called fluid-phase endocytosis, is an actin-dependent process, which involves the internalization of large amounts of extracellular fluid (rather than particulates) through extensions of the plasma membrane. Alternatively, aggregates of ADCs could be taken up by phagocytosis, implying deformations in the cell membrane (created by rearrangement of actin) that engulf the particle. High drug antibody ratio (DAR), which imparts significant hydrophobicity to ADCs, may also increase their uptake in tumor cells. However, this unspecific endocytic capacity can also occur in Kupffer cells or hepatic sinusoidal endothelial cells, accelerating ADCs clearance [24] and potentially contributing to systemic toxicity.

### Ab

After the molecular target recognition, the selection of the appropriate Ab plays a key role in pharmacokinetic/ pharmacodynamic profiles and therapeutic index [15]. In addition to high binding affinity to the tumor cell-surface antigen, an ideal monoclonal Ab should have low immunogenicity, low cross-reactivity, and long plasma half-life. The monoclonal Ab used to construct ADCs is generally of the immunoglobulin G (IgG) class (~150 kDa) that consists of two heavy or H chains (of approximately 50 kDa) and two light or L chains (of 25 kDa). Heavy and light chains are linked to each other by disulfide bonds. The antigen-binding activity is mediated by the antigen-binding fragments (Fabs), whilst the binding of the Ab with effector cells of the immune system and the regulation of IgG half-life in circulation by binding to the neonatal constant fragment (Fc) receptor (FcRn) is mediated by the Fc. The IgG1 subtype is the most frequently used in ADCs design thanks to its serum stability.

As already mentioned, humanized or fully human Abs are used to overcome the immunogenicity observed with the first murine-based ADCs. Chimeric Abs are created by fusing domains from different species (e.g., the entire variable region from mouse or rabbit with the constant domain of human origin). The FDA-approved brentuximab vedotin has an Ab of this type. In contrast, humanized Abs contain segments of foreign-derived amino acids grafted into human-derived Fab regions and constant regions. Hence, they are less immunogenic and represent a safer choice for clinical development. Polatuzumab vedotin, gemtuzumab ozogamicin, trastuzumab emtansine, belantamab mafodotin, trastuzumab deruxtecan, and sacituzumab govitecan are FDA-approved ADCs based on humanized Abs. In fully human Abs, no part is mouse-derived, providing a lower incidence of the immune response compared to humanized counterparts: an example is the FDA-approved enfortumab vedotin.

The THIOMAB Ab engineering-based method allows the incorporation of reactive cysteine substitutions in the Ab Fab region itself, producing more homogeneously loaded ADCs, with precise control over site reactivity and DAR. It preserves interchain disulfide bridges and conjugates efficacy, and it has been used for various ADCs, including the FDA-approved polatuzumab vedotin [25–28].

### Linker

Linkers, through which the bond between the drug and the Ab is created, are maybe the most crucial and complex components in the construction of a successful ADC [15, 29–31]. Their chemistry, stability, and mode of conjugation are essential to avoid unwanted release of the drug in the blood circulation and be readily cleaved when internalized in the cancer cell to release the payload only at the target site. Furthermore, hydrophobicity is another criterion to be considered since hydrophobic linkers increase the risk of aggregates of ADC molecules, which could then get rapidly cleared by the liver and act as immunogenic substances. Thus, linkers are crucial in determining pharmacokinetics, the pharmacodynamics, and therapeutic window of ADCs. The currently FDA-approved ADCs use two classes of linkers, which differ in the payload release mechanism: non-cleavable and cleavable linkers.

Non-cleavable linkers consist of non-reducible bonds with the amino acid residues of the Ab that resist proteolytic degradation, have improved plasma stability and reduced off-target toxicity in comparison to cleavable linkers. After ADC internalization, non-cleavable linkers require the complete degradation of the Ab moiety by cytosolic and lysosomal proteases to liberate the cytotoxic drug linked to an amino acid residue derived from the degraded Ab [14]. Generally, non-cleavable linkers are formed by thioether or maleimidocaproyl groups. FDA-approved ADCs using non-cleavable linkers are trastuzumab emtansine, with a thioether linker (*N*-succinimidyl-4-(*N*-maleimidomethyl) cyclohexane-1-carboxylate, and belantamab mafodotin, with a protease-resistant maleimidocaproyl linker.

Cleavable linkers are designed either for internalizing and not internalizing ADCs since the release of the drug is sensitive to the extracellular and intracellular environment differences (e.g., pH and redox potential) or to specific lysosomal enzymes (such as cathepsin B). There are four most used cleavable linkers: hydrazone linkers, cathepsin B-sensitive linkers, glutathione (GSH)-sensitive disulfide linkers, and β-glucuronidase-sensitive linkers. ADCs based on cleavable linkers are more likely to have the already-mentioned by-stander effect, which can affect also tumor cells with no or lower expression of the target but also normal cells present in the tumor microenvironment. Albeit at possibly lower levels, similar effects are also expected to occur with non-cleavable linkers due to release of payloads from dead cells.

The hydrazone linkers bear acid-sensitive or acid-labile chemical bonds that ensure a pH-dependent drug release. The hydrazone portion is formed by the condensation of a hydrazine with aldehydes or ketones, thus producing a C=N bond that is stable at physiological conditions (pH 7.4 in the bloodstream) and hydrolyzed once transported in the acidic cellular compartment such as the lysosome (pH < 5) or late endosomes (pH 5.5–6.2). A hydrazone linker is a component of the pioneering ADC gemtuzumab ozogamicin [32], approved in 2000 but withdrawn from the market in 2010 due to a lack of clinical benefit and low tolerability.

The cathepsin B-sensitive linkers are based on the carboxydipeptidase activity of the lysosomal cysteine protease cathepsin B, involved in tumor progression and overexpressed in various cancer cells. Cathepsin B has a broad spectrum of substrates but preferentially recognizes sequences such as Val-Cit, phenylalanine (Phe)-lysine (Lys), and Val-Ala [33, 34]. The protease breaks the dipeptide bond on the C-terminal side, promoting an enzymatic drug release from the ADC once internalized. Furthermore, peptide-based linkers are more stable than acid-based linkers in unsuitable pH and in the plasma due to the presence of protease inhibitors. To date, peptide sequences that mimic the substrate for a protease have been used in many ADC designs. One of the so far most successful linkers is the cleavable Val-Cit linker, used, among others, in brentuximab vedotin [30, 35]. Maleimide-based drug conjugation is the method of choice for the construction of cathepsin B-sensitive linkers. The conjugation between maleimides and cys residues on the Ab creates a sulfur bond that leads to a thiosuccinimide adduct. However, the bond is only moderately stable and can easily undergo slow hydrolysis or deconjugation by inverse Michael addition reaction with reactive thiols in plasma. This degradation results in payload transfer loss, with consequent efficacy reduction and increased toxicity, and the substitution of conventional endocyclic maleimides derivatives with the exocyclic ones is a strategy for the preparation of fully thiol-exchange resistant products [36].

In 2012, tesirine was designed as a drug-linker to combine the potent anticancer activity of the PBD DNA cross-linker warhead SG3199 (see below) with desirable pharmacokinetic properties (e.g., low hydrophobicity contributing to low aggregation) and improved conjugation characteristics. Hence, one of the reactive imines in its carbinolamine form was bond to the cathepsin B-cleavable Val-Ala dipeptide linker and to a further uncharged polyethylene glycol (PEG-8) spacer that increases ADC mobility and solubility [37]. Furthermore, fast and straightforward conjugation to the monoclonal Ab cysteine was obtained by maleimide Michael addition. Tesirine is the drug-linker component of ADCs under evaluation in over 15 clinical trials, in either solid or hematological tumors [38].

The GSH-sensitive disulfide linkers are cleaved in tumor cells due to the high concentration of GSH. The latter is a low molecular weight thiol, and its low concentration in the extracellular environment (about 5 μmol/L in blood) determines the stability of GSH-sensitive linkers in the blood flow [39]. The disulfide bond between Ab and drug is thermodynamically stable at physiological pH in the systemic blood stream but sensitive to nucleophile attack by thiols. As mentioned, the release strategy relies on the higher concentration of reducing GSH in the cytoplasm (1–10 mmol/L) and the marked difference in reduction potential that the ADC encounters once internalized. Abundant intracellular GSH reductively breaks the disulfide linker, releasing the payload part of the ADC. Importantly, tumor cells often present an oxidative stress, which further increases GSH levels: this contributes to the specificity of ADCs for release of their payloads in cancer cells rather than in healthy cells. To further enhance the stability of the bond while the ADC is in circulation, the disulfide linkers are often associated with methyl groups that act as hinderance groups near the disulfide cleavage sites, such as in the disulfide *N*-succinimidyl 4-(2-pyridyldithio)pentanoate (SPP) linker containing a single methyl group or SPDB having two methyl groups [10, 40].

Finally, the β-glucuronidase-sensitive linkers are protease-sensitive linkers hydrolyzed by β-glucuronidase for the payload release. β-glucuronidases are hydrolytic enzymes widely diffused in lysosomes and tumor necrotic regions that degrade β-glucuronic acid residues into polysaccharides. The specific site of action of β-glucuronidase ensures the stability of the ADC in the blood flow and induces a selective release of the drug [41, 42].

### Payload

Ideal payload for an ADC should have high stability in the blood flow and lysosomes, *in vitro* subnanomolar half maximal inhibitory concentration (IC_50_), low immunogenicity, long half-life, small molecular weight, good solubility in aqueous environment of Ab, and functional groups to facilitate conjugation to the Ab maintaining the internalization property of the ADC. An average of two to four highly potent cytotoxic molecules are bound to each Ab and this represents the already mentioned DAR.

**Figure 1.**
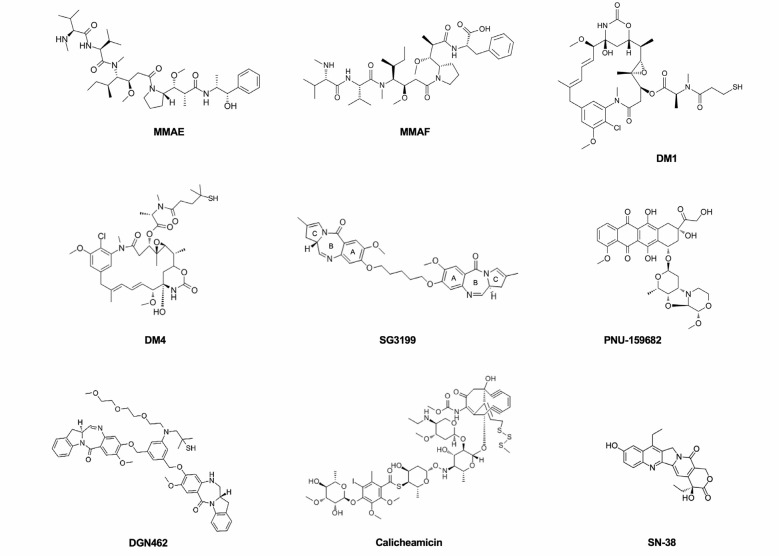
The principal payloads used in ADCs production, belonging to the drug classes of microtubule-disrupting agents and DNA-targeting agents

Microtubule-disrupting agents and DNA-targeting agents are currently the classes of payloads mostly widely used in ADC design (Figure 1). Tubulin inhibitors exploit the more rapid cellular proliferation of cancer cells compared to normal cells [43, 44]. They are grouped into two main categories depending on their mechanism of action. One is represented by microtubule-stabilizing agents that prevent the depolymerization of microtubules and enhance their polymerization, leading to more stable and less functioning filaments. The second one comprises microtubule-destabilizing agents that impede tubulin assembly and the formation of mature microtubules. Vinca alkaloids, taxanes, auristatins, maytansinoids, cryptophycins, hemiasterlin, and discodermolide are the main tubulin inhibitors that have been used and investigated for their possible use in ADC construction.

**Figure 2.**
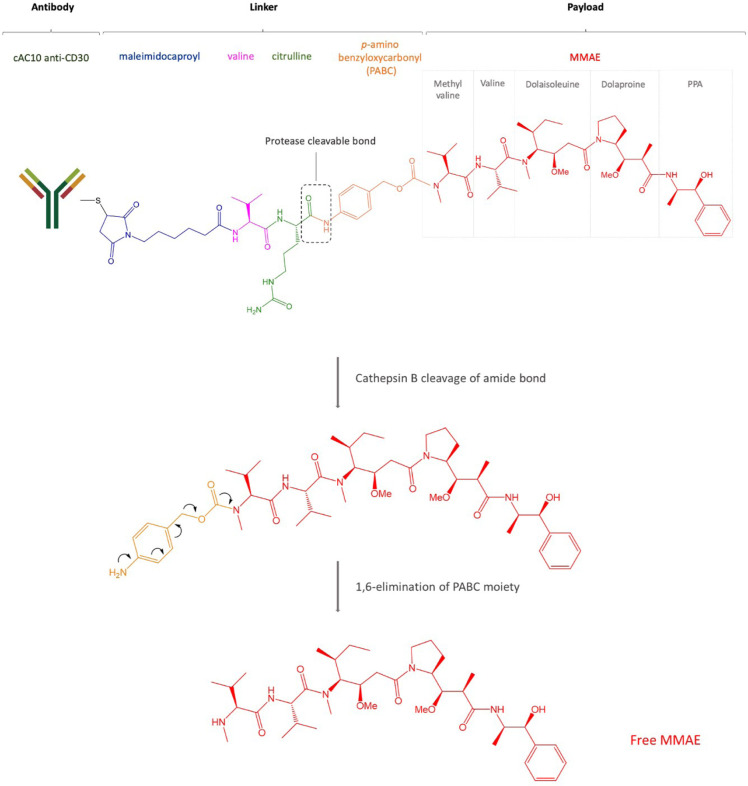
Structure of brentuximab vedotin. After internalization, the payload MMAE binds microtubules, prevents polymerization, and induces cell death

Among the most so-far commonly used payloads, MMAE is a microtubule-disrupting agent belonging to the class of auristatins, which inhibit cell division by blocking the polymerization of α- and β-tubulin monomers, thus inducing apoptosis. The binding region is located at the interface between the β1-tubulin and the adjacent α2-tubulin subunit of two longitudinally aligned tubulin dimers. *In vitro* results suggest that the bond of auristatins to tubulin creates curved spiral or ring-shaped aggregates that alter the normal function of microtubules. Similar curved structural conformation of microtubules is induced by the vinca alkaloids such as vincristine or vinblastine but also by cryptophycin and dolastatins [45]. These are synthetic analogues of dolastatin 10, a pentapeptide derived from the sea hare *Dolabella Auricularia* which failed to show an appreciable therapeutic index in clinical trials because of significant toxic side effects. MMAE is 100–1,000 times more potent than its precursor and differs from the structure of dolastatin 10 only for the C-terminal in which the dolaphenine is substituted by (1*S*,2*R*)-(+)-norephedrine (PPA). Although it has a potent antiproliferative effect on tumor cells, the lack of specificity impedes its direct use as an anti-tumor drug. The MMAE structure is composed of five peptide residues: monomethyl valine (MeVal), Val, dolaisoleuine (Dil), dolaproine (Dap), and the carboxy-terminal amine PPA (Figure 2). Based on the partially hindered rotation around the Dap-Dil amide bond, two different conformational isomers can be distinguished: *cis*-conformer and *trans*-conformer. Although the isomers have an equal proportion, only the *trans*-conformer is biologically active [46, 47].

The MMAF is another common payload, which, as well as other auristatin derivatives, binds to tubulin and inhibits its polymerization, inducing G2/M arrest and apoptosis in the targeted cells. MMAF differs from its analogue MMAE for a Phe moiety at its C-terminus which confers more hydrophilicity, lower systemic toxicity, and higher IC_50_ [48].

Maytansinoids are chemical derivatives of the naturally occurring maytansine, originally isolated in 1972 from the bark of the African shrub *Maytenus ovatus*. Maytansine was one of the first compounds to show picomolar IC_50_ activity and higher potency (100-fold to 1,000-fold) than doxorubicin or paclitaxel but due to a narrow therapeutic window associated with high systemic toxicity, it was substituted by its more effective derivatives. Maytansinoids are 19-member macrocyclic lactams attached to a chlorinated benzene and differ from their precursor in the substituents on the ester group at C3. In fact, structure activity relationship (SAR) studies showed that the C3 ester is essential for antiproliferative effect but variability in the side chain is allowed. The C9 carbinolamine is another structural feature required for activity, which, instead, is diminished in the case of etherification in this position. The *N2*’-deacetyl-*N2*’-(4-mercapto-4-methyl-1-oxopentyl) (DM4 or ravtansine) and the other maytansinoids are anti-tubulin agents that bind to tubulin at or near the vinblastine-binding site, thus interfering with the microtubule dynamic and causing apoptosis due to mitotic arrest. The *N2*’-deacetyl-*N2*’-(3-mercapto-1-oxopropyl)-maytansine (DM1 or mertansine) is a member of the maytansinoid family used as payload.

DNA-damaging agents can kill cancer cells at any point of their cell cycle with at least four different mechanisms of action: DNA double-strand breakage, DNA alkylation, DNA intercalation, and DNA cross-linking. The most used DNA-damaging payloads are calicheamicins, duocarmycins, PBDs, doxorubicin, and camptothecin analogues. Calicheamicins are potent antitumor antibiotics, which owe their name to the first derivative of this class, originally isolated from the fermentation broth of actinomycete *Micromonospora echinospora*. They recognize the minor groove of the TCCTAGGA sequence of DNA, causing double-strand breaks that induce apoptotic cell death [49]. To increase drug stability and to improve its therapeutic effect, calicheamicin structure was optimized by acetylation of the aminosugar and by conversion to the more stable disulfide derivative *N*-acetyl-γ-calicheamicin dimethyl hydrazide, the payload used in the CD22 targeting inotuzumab ozogamicin (see below). Its complex structure consists of a trisulfide portion, four carbohydrate residues, a hexasubstituted benzene ring, an N-O glycosidic linkage, and a bicyclo[7.3.1]tridec-9-ene-2, 6-diyne system. The aryl-tetrasaccharide domain is believed to be the principal responsible for inducing double-strand DNA breaks, which represent the mechanism of action. The N-O bond orients the molecule into a shape suitable for interacting with the minor groove of DNA and the iodo group of the saccharide portion binds the 2-amino group of 5’-deoxyguanosine, providing stability. The reduction of calicheamicins by cellular thiols generates a 1,4-dehydrobenzene diradical that abstracts hydrogen atoms from duplex DNA, thereby causing double-strand cleavage. The tetranucleotide pyrimidine tracts TCCT and CTCT seem to be prominent binding and cleavage sites [50].

Duocarmycins are DNA minor groove-binding alkylating agents that disrupt the nucleic acid architecture in the A-T-rich region [51, 52]. Doxorubicin is a DNA intercalating agent that inhibits DNA synthesis and camptothecin analogs, such as topotecan, irinotecan, SN-38, and DX-8951f, are inhibitors of the nuclear enzyme topoisomerase I [53, 54]. PBDs, for example, SG3199, form single alkylated DNA adducts binding to guanine residues in the DNA minor groove [55]. Discovered in 1963 after the extraction of anthramycin (ANT) from the thermophilic actinomycete *Streptomyces refuineus*, PBDs are tricyclic systems that consist of an anthranilate ring, a 1,4-diazepin-5-one ring, and a pyrrolidine ring. Compounds of this class differ from one another by the substituents in the anthranilate- and pyrrolidine-rings, in addition to the position of unsaturation in the pyrrolidine-ring [56]. PBDs are DNA crosslinkers or alkylators whose mechanism of action depends on the formation of covalent bonds with the exocyclic amino group of the guanine base at preferred GATC sequences in the minor groove of DNA. The N2 of the guanine and the C11 position of PBD are involved in the nucleophilic attack. The resulting PBD-DNA adducts, which persisted for 36 h, block DNA strand separation, thus inhibiting essential DNA metabolic processes such as replication and inducing apoptosis at low nanomolar to picomolar concentrations. The use of PBD dimers, such as SG3199, ensures two covalent bonds with guanine bases and to span greater lengths of DNA via cross-linking [37]. Furthermore, PBD dimers differ from conventional DNA crosslinking agents (nitrogen mustards or platinum drugs) because they cause moderate distortion of the DNA structure, thus evading the DNA damage repair responses [57]. Compared to early generation of dimerized PBD warheads, SG3199 includes a longer 5’-carbon linker between the two PBD monomers that induces a > 3,400-fold increase in cytotoxicity and > 10-fold DNA cross-linking ability [58].

Targeted agents such as BCL2 inhibitors, spliceosome inhibitors, and transcription inhibitors targeting RNA polymerase II are among payloads with different mechanisms of actions [6, 16, 59–62].

## Target, clinical efficacy and side effects of available ADCs

In this section, we will discuss mechanism of action, efficacy, and main side effects of clinically investigated ADCs in the lymphoma setting (Table 2). It is worth mentioning that we can define two types of ADC-related toxicities [4, 6, 63]. Some can be “on-target”, attributed to a cumulative effect of cytotoxic agent in non-tumoral tissues expressing the targeted antigen, however, most AEs are generally driven by payload systemic release namely “off-target” AEs. For instance, MMAE-sharing ADCs such as brentuximab vedotin, polatuzumab vedotin, and enfortumab vedotin (an ADC used to treat bladder cancer), all can cause peripheral neuropathy (off-target AE); on the other hand, only enfortumab vedotin appears to be associated to dysgeusia, an AE likely to be related to its antigen-target (Nectin 4), expressed within the salivary glands (“on-target” AE). IRR is a frequent AE shared by all ADCs. Key AEs of ADCs are summarized in Table 2.

### CD30

CD30 is a transmembrane glycoprotein, a member of the tumor necrosis factor (TNF) superfamily and it is expressed in activated B and T cells, and, commonly, in HL, ALCL, and a small proportion of B cell lymphomas [64]. Brentuximab vedotin is an ADC based on an anti-CD30 mouse/human chimeric Ab (cAC10, SGN-30), obtained from the fusion of the variable heavy and light region of the murine anti-CD30 Ab AC10 with the constant γ1-heavy and λ-light region of the human IgG1 (Figure 1, Table 2). Interestingly, the Ab itself induces growth arrest of CD30^+^ cell lines [65] but brentuximab vedotin is up to 340-fold more active than the naked Ab [66]. The cytotoxic payload of brentuximab vedotin is the microtubule-disrupting agent MMAE. Even if each Ab could bind a maximum of eight MMAE molecules due to the presence of eight cysteines, brentuximab vedotin carries an average of four payload molecules to avoid the higher *in vitro* cytotoxicity shown by the same ADCs loaded with more drugs [67]. The brentuximab vedotin linker system between the Ab and MMAE was designed to be stable in the blood flow and consists of a maleimidocaproyl spacer, the protease-sensitive dipeptide Val-Cit, and the self-immolative PABC moiety, a spacer between the dipeptide and the drug. The latter possesses a self-cleavage ability and provides an easier access to cathepsin B for its cleavage sequence. The linker is bound to the Ab through a thioether bond between the terminal thiol of cysteine residues of the Ab heavy and light chains and the maleimide portion. The protease cleavage of Cit-PABC amide bond creates an instable PABC-substituted MMAE intermediate that undergoes a spontaneous 1,6-elimination, generating *p*-aminobenzyl alcohol, CO_2_, and free molecules of MMAE (Figure 1) [30].

In August 2011, the anti-CD30 ADC brentuximab vedotin obtained accelerated approval for the treatment of relapsed HL after that phase 2 studies in HL patients relapsing after autologous hematopoietic stem cell transplantation (auto-HSCT, *n* = 102] and in systemic ALCL (*n* = 58) showed high overall response rates (ORRs; HL, 75%; ALCL, 86%) and complete response (CR) rates (HL, 34%; ALCL, 33%) [68–70]. Importantly, in a similar population of HL patients, the recently published KEYNOTE-204 phase 3 trial (NCT02684292) has demonstrated a superior activity of the anti-PD1 Ab pembrolizumab than brentuximab vedotin: median progression-free survival (PFS), 13 months *vs.* 8 months; hazard ratio (HR), 0.65 [71].

In 2015, brentuximab vedotin was approved for patients with classical HL at high risk of relapse or progression as post auto-HSCT consolidation. This is based on the AETHERA phase 3 trial (NCT01100502) showing improved PFS in patients receiving brentuximab vedotin (*n* = 165) or placebo (*n* = 164) after auto-HSCT: HR, 0.57; median PFS, 42.9 months *vs.* 24.1 months; 2-year PFS, 63% *vs.* 51% [72]. The latter appeared maintained with a longer follow-up (5-year PFS, 59% *vs.* 41%) [73].

In 2017, FDA extended the approval of brentuximab vedotin to patients with primary cutaneous ALCL (cALCL) or CD30-positive MF who have received prior systemic therapy. This was based on the ALCANZA phase 3 trial (NCT01578499) results [74, 75]. In this study, adult patients with previously treated CD30-expressing MF/cALCL were randomly assigned 1:1 to either brentuximab vedotin (*n* = 64) or physician’s choice of methotrexate or bexarotene (*n* = 64). The ADC-containing arm was superior to the physician’s choice arm, achieving a higher percentage of objective responses lasting at least 4 months (56.3% *vs.* 12.5%, *P* < 0.001) and an improved CR rate (17.2% *vs.* 1.6%, *P* = 0.002). With a median follow-up of 46 months, a significant benefit in median PFS also became evident (16.7 months *vs.* 3.5 months, *P* < 0.001) [74, 75].

In 2018, brentuximab vedotin was FDA-approved for the treatment of adult patients with previously untreated stage III or IV classical HL in combination with chemotherapy based on the ECHELON-1 phase 3 trial (NCT01712490), in which 664 patients were randomized between brentuximab vedotin, doxorubicin, vinblastine, and dacarbazine (BV-AVD) and 670 to doxorubicin, bleomycin, vinblastine, and dacarbazine (ABVD) [76]. The BV-AVD regimen was shown superior to ABVD (2-year modified PFS, 82% *vs.* 77%; HR, 0.77) [76], a result confirmed with a longer follow-up (5-year PFS, 82% *vs.* 75%; HR, 0.68) [77]. The same year, brentuximab vedotin was approved in combination with chemotherapy for adult patients with untreated systemic ALCL or another CD30-positive PTCL, based on the ECHELON-2 phase 3 trial (NCT01777152) [78, 79]. In this trial, patients with CD30-positive PTCL were randomized between a treatment with brentuximab vedotin, cyclophosphamide, doxorubicin, and prednisone (BV-CHP) or standard cyclophosphamide, doxorubicin, vincristine, and prednisone (CHOP) regimen [79]. The ADC-containing arm (*n* = 226) was superior to the chemotherapy-only arm (*n* = 226): HR, 0.71; median PFS 48 months *vs.* 21 months; 3-year PFS, 57% *vs.* 44%; ORR, 83% *vs.* 72%; CR, 68% *vs.* 56% [79].

In April 2022, six phase 3 trials (five in HL patients) are further defining the role of brentuximab vedotin. The SWOG S1826 (NCT03907488) trial is comparing the already mentioned BV-AVD regimen against the AVD combined with an anti-PD1 Ab nivolumab (N-AVD) instead of the ADC in both pediatric and adult patients with newly diagnosed advanced stage HL [80]. The AHOD1331 trial (NCT02166463) is comparing a scheme with brentuximab vedotin, doxorubicin, vincristine sulfate, etoposide, prednisone, and cyclophosphamide (BV-AVEPC) *vs.* doxorubicin, bleomycin, vincristine, etoposide, prednisone, and cyclophosphamide (ABVE-PC) in children and young adults with stage IIB or stage IIIB-IVB HL. The RADAR trial (NCT04685616) has been designed to randomize untreated stage IA/IIA HL patients to receive either ABVD chemotherapy or the brentuximab vedotin containing AAVD regimen (doxorubicin, brentuximab vedotin, vinblastine, and dacarbazine). Treatment will be adapted based on the results of an interim PET-CT scan performed after 2 cycles of treatment (PET2). Patients will receive one further cycle of their randomized chemotherapy if they are able to achieve a complete metabolic response at interim PET-CT scan (Deauville score 1–3), two additional cycles if achieving a partial metabolic response (Deauville score 4) or will get further treatment at their treating clinician’s discretion if they experienced an insufficient response or progressive disease (Deauville score 5). The HD21 trial (NCT02661503) randomly assigned untreated advanced stage HL patients to BrECADD (brentuximab vedotin, etoposide, cyclophosphamide, doxorubicin, dacarbazine, dexamethasone) or to escalated BEACOPP (bleomycin, etoposide, doxorubicin, cyclophosphamide, vincristine, procarbazine, prednisone), each followed by consolidation radiotherapy to PET-positive residual lesions. Based on the feasibility and activity (CR, 61%, ORR, 82%) reported in a phase I/II trial [81], the combination of nivolumab and brentuximab vedotin is being compared to the ADC alone in relapsed or refractory (R/R) HL patients (CheckMate 812, NCT03138499). Finally, following phase I study (ORR, 53%; CR, 41% in the first 17 enrolled patients) [82], an ongoing study is comparing lenalidomide combined with brentuximab vedotin or with rituximab in R/R DLBCL patients stratified by CD30 expression (≥ 1% *vs.* < 1%, NCT04404283) [83].

Brentuximab vedotin is also still under evaluation in many early-phase trials. Examples are studies exploring BV R-mini-CHP regimen (brentuximab vedotin, rituximab, cyclophosphamide, doxorubicin, vincristine, prednisone) regimen as the first line for elderly DLBCL patients [84], BV-AVD as the first line for PTCL patients other than systemic ALCL with low CD30 expression [85], BV-ICE (brentuximab vedotin, ifosfamide, carboplatin, etoposide) for R/R HL patients [86], brentuximab vedotin plus the HDAC inhibitor romidepsin for systemic CTCL patients requiring therapy [87]. New anti-CD30 ADCs are also under development [88–93], such as F0002-ADC [94] currently being investigated in a phase I study (NCT03894150, Table 2).

### CD79B

CD79B is a transmembrane protein, along with CD79a and a surface immunoglobulin, forms the B cell receptor (BCR) complex. Expressed on the surface of over 90% B cell lymphomas, CD79B is a valid target to selectively deliver payloads to B cell lymphomas [21, 64, 95]. Polatuzumab vedotin contains an anti-CD79B humanized IgG1 monoclonal Ab, which is, similarly to brentuximab vedotin, covalently conjugated to the microtubule-disrupting anti-mitotic agent MMAE (Table 2) [28]. The linker is a maleimidocaproyl-Val-Cit-PABC protease-cleavable peptide. The average DAR for polatuzumab vedotin is 3.5 molecules of MMAE attached to each Ab.

Polatuzumab vedotin has been the second ADC to receive FDA approval for the treatment of lymphoma patients, namely adults with R/R DBCL in combination with bendamustine and rituximab (BR) after at least two prior therapies. The accelerated approval (June 2019) was based on a phase Ib/II trial (NCT02257567) evaluating safety, tolerability, and activity of the ADC R/R follicular lymphoma (FL) or DLBCL that showed a CR rate of 40% with polatuzumab vedotin plus BR compared to 18% with BR alone and an ORR of 63% with polatuzumab vedotin plus BR compared to 25% with BR [96, 97]. Additionally, the median overall survival (OS) in patients who received polatuzumab vedotin plus BR was higher compared to patients who received BR alone (12.4 months *vs.* 4.7 months) [96].

Six phase 3 trials are investigating the role of polatuzumab vedotin in aggressive B cell lymphoma, including two studies as the first line for DLBCL. The POLARIX phase 3 trial (NCT03274492) comparing a standard R-CHOP regimen (rituximab, cyclophosphamide, doxorubicin, vincristine, and prednisone) against a regimen replacing the tubulin-targeting agent vincristine with polatuzumab vedotin as a frontline for DLBCL patients (pola-R-CHP) recently met its primary endpoint demonstrating a significant 6.5% difference in 2-year PFS benefit [98]. The POLAR BEAR trial, focusing on elderly DLBCL patients, is comparing R-mini-CHOP against R-mini-CHP + polatuzumab vedotin in the first line setting (NCT04332822). The other phase 3 trials are all devoted to R/R DLBCL, associating polatuzumab vedotin to different salvage chemotherapy regimens: i) rituximab, ifosfamide, carboplatin, and etoposide plus (Pola-R-ICE, NCT04833114); ii) rituximab, gemcitabine and oxaliplatin (Pola-R-Gemox, NCT04182204); and iii) rituximab bendamustine studied in a Chinese population (NCT04236141). Furthermore, polatuzumab vedotin is the subject of a series of on-going phase I/II trials. Promising preliminary results without unexpected toxicities have been so far presented for the combination with rituximab and lenalidomide (NCT02600897) [99], obinutuzumab and lenalidomide (NCT02600897) [100], mosunetuzumab (NCT03671018) [101], rituximab and venetoclax (NCT02611323) [102], obinutuzumab and venetoclax (NCT02611323) [103]. It is worth mentioning that in a retrospective study, polatuzumab vedotin in combination with rituximab has shown limited and short-lasting activity in R/R lymphoma patients after failure of anti-CD19 chimeric antigen receptor (CAR) T cell therapy [104].

Among other ADCs targeting CD79B (Table 2) [105, 106], iladatuzumab vedotin evaluated alone or in combination with rituximab in a phase I study in patients with R/R B cell lymphomas or chronic lymphocytic leukemia (CLL) demonstrated an unsatisfactory ORR and safety profile that hindered its progress into phase II trials (NCT02453087) [107]. To our knowledge, no clinical data are available for additional CD79B ADCs.

### CD19

CD19 is a 95 kDa transmembrane glycoprotein, whose expression is induced during the differentiation of the hematopoietic stem cell of the B lymphocyte lineage and is maintained in preB and mature B cells until their terminal differentiation into plasma cells [64]. Although CD19 is the most ubiquitously expressed protein in normal B-lineage cells, malignant cells rarely lost CD19 antigen during the process of neoplastic transformation and maintain its high-level expression [80% of acute lymphocytic leukemia (ALL), 88% of B cell lymphomas, and 100% of B cell leukemias]. Furthermore, except for the B cell lineage, CD19 is expressed neither in other normal tissues [108, 109] nor in hematopoietic stem cells, hence immunotherapy can be selectively directed to this target without affecting the repopulation of B cell compartment [110]. These properties make CD19 not only a useful marker in the diagnosis of leukemias and lymphomas but also a valuable therapeutic target for the development of ADCs against B cell malignancies [21]. Several ADCs composed of a covalent association of an anti-CD19 monoclonal Ab with chemically different payloads have shown promising results for the treatment of lymphoma both in preclinical and clinical studies (Table 2) [111–116]. Among them, four ADCs targeting CD19 are currently in clinical development: coltuximab ravtansine (SAR3419, huB4-DM4), loncastuximab tesirine (ADCT-402), and denintuzumab mafodotin (SGNCD19A).

Conjugation of the humanized monoclonal IgG1 anti-CD19 Ab (huB4) to the potent cytotoxic drug DM4 generated the ADC coltuximab ravtansine. The monoclonal Ab is chemically linked to the payload via the cleavable disulfide cross-linking agent SPDB, an uncharged, lipophilic linker that binds the monoclonal Ab through the formation of amides with the ε-amino group of Lys. At the other end, the available thiol-reactive group of SPDB is conjugated with the terminal sulfhydryl function of maytansinoid DM4. The DAR for coltuximab ravtansine is ~3.5 maytansinoids for each monoclonal Ab. Studies on the intracellular trafficking of coltuximab ravtansine suggest that after internalization via endocytosis, the Ab portion is fully degraded in lysosomes, giving the initial Lys-*N*-SPDB-DM4 metabolite. The subsequent reduction of the disulfide bond cleavage between linker and drug, presumably made by GSH, generates the non-charged, membrane-permeable free species of the payload. The free thiol species of DM4 are then converted in the *S*-methyl-DM4 form by cellular methyl transferases, which contribute to tumor eradication via bystander effect against neighboring tumor cells [117].

With favorable antitumor activity and preclinical results in lymphomas [111], coltuximab ravtansine has been explored in early phase trials either as a monotherapy or in combination with rituximab in R/R DLBCL (NCT01472887, NCT01470456) demonstrating an acceptable safety profile, however, showing only modest clinical activity [118, 119].

Loncastuximab tesirine (ADCT-402) is a more successful CD19 ADC, in which the Ab is stochastically conjugated through a cathepsin-cleavable linker to the PBD dimer SG3199. As above-mentioned, the linker-drug portion is also referred as tesirine or SG3249 (Table 2). Loncastuximab tesirine is not cleaved into the blood flow and has a rapid internalization kinetics, after which it is trafficked to lysosomes and processed to release the drug [113].

*In vitro* and *in vivo* experiments showed remarkable efficacy as a single agent in CD19^+^ lymphoma and leukemia models [113, 120] and an increased potency of the PBD dimer compared to ADCs delivering tubulin inhibitor drugs [113]. Synergism at preclinical level has also been observed combining loncastuximab tesirine with venetoclax, with phosphoinositide 3-kinase (PI3K) inhibitors (idelalisib, copanlisib), Bruton tyrosine kinase (BTK) inhibitors (ibrutinib), and with bendamustine [120].

Loncastuximab tesirine shows promising single agent activity in several early phase I/II trials [57, 121–123] across different B cell malignancies and has been approved on April 2021 by the FDA based on the results of the phase 2 LOTIS-2 study (NCT03589469) [123], for adult patients with R/R large B cell lymphoma after at least two prior lines of systemic therapy. Side effects are mostly represented by neutropenia, thrombocytopenia, anemia, fatigue, nausea, edema, and liver enzyme abnormalities [57, 121, 123]. Loncastuximab tesirine has been evaluated in combination with durvalumab, an anti-PDL1 Ab in a phase Ib in R/R DLBCL, MCL, or FL, showing no clear benefit (NCT03685344) [124] and it is currently evaluated for R/R B cell lymphomas in combination with other agents, such as rituximab (NCT04998669), ibrutinib (NCT03684694) [125], idelalisib (NCT04699461) [126], venetoclax (NCT05053659), with R-BAC immunochemotherapy, rituximab-bendamustine, Ara-C in MCL (NCT05249959) and with gemcitabine, or lenalidomide, or the PI3Kδ/CK1ε inhibitor umbralisib or polatuzumab vedotin in a large multi-arm phase I trial for R/R B cell lymphoma patients (LOTIS-7 study, NCT04970901). Its efficacy is also explored in Waldenström macroglobulinemia (NCT05190705).

Early results are so far available only for the first 36 R/R DLBCL or MCL patients treated with the ADC plus ibrutinib in the LOTIS-3 1/2 study (NCT03684694). With a manageable toxicity, the combination led to an ORR of 67% in non-GCB DLBCL, 20% in GCB DLBCL, and 86% in MCL [127]. Additionally, a phase 3 trial (LOTIS-5, NCT04384484) is comparing loncastuximab tesirine plus rituximab against R-GemOx (rituximab, gemcitabine, and oxaliplatin) for R/R DLBCL patients [128], while a phase 2 is testing loncastuximab tesirine plus rituximab as 1st line for unfit/frail DLBCL patients (LOTIS-9, NCT05144009). An initially planned phase I study (LOTIS-8, NCT04974996) exploring the addition of loncastuximab tesirine to R-CHOP as 1st line for unfit/frail DLBCL patients has been instead withdrawn.

Denintuzumab mafodotin is CD19 targeting ADC that incorporates, via a non-cleavable maleimidocaproyl linker, the tubulin targeting MMAF as payload (Table 2). Denintuzumab mafodotin was studied as a single agent in two phase 1 trials respectively for patients with B cell ALL or highly aggressive B cell lymphoma (NCT01786096) [129] and with R/R B cell lymphoma (NCT01786135) [130]. In the latter trial, denintuzumab mafodotin demonstrated encouraging anti-tumor activity with 33% of patients achieving objective responses, including 22% (13/60) with CRs and a generally well-tolerated safety profile [130]. Two phase 2 clinical trials aiming to explore its feasibility and efficacy in combination with other chemotherapeutic agents: i) R-ICE (rituximab, ifosfamide, carboplatin, and etoposide) in R/R DLBCL, lymphoblastic leukemia and FL (NCT02592876); ii) with R-CHOP or R-CHP as front-line therapy in patients with DLBCL or grade 3b FL (NCT02855359) have been prematurely terminated based on sponsor decision.

### CD22

CD22 is a 135 kDa type I transmembrane glycoprotein, belonging to the sialic acid-binding immunoglobulin-like lectin receptor family. It is a B cell lineage-specific differentiation antigen, expressed on immature and mature B cells and lost upon terminal differentiation to plasma cells [64]. Recent studies suggest that it regulates B cell functions, such as survival, via BCR activation and acts as an adhesion molecule. CD22 is also upregulated in B cell tumors including most lymphomas and many leukemias, hence it is an ideal target for the ADC therapeutic approach [21, 131].

Among the anti-CD22 ADCs (Table 2) [132–140], inotuzumab ozogamicin (CMC-544) was approved by the FDA in August 2017 for R/R B cell ALL as monotherapy and it is also increasingly evaluated in preclinical and clinical trials against lymphoma. Inotuzumab ozogamicin is composed of recombinant, humanized IgG4 kappa CD22-directed monoclonal Ab covalently linked via an acid-labile 4-(4’-acetylphenoxy) butanoic acid linker to *N*-acetyl-γ-calicheamicin dimethyl hydrazide, the above-discussed semi-synthetic derivative of calicheamicin. The average loading of the drug is 2.5 mol of calicheamicin per mole of Ab [141]. After binding to CD22, the ADC is rapidly internalized into the lysosomal compartment, where the cleavable hydrazone linker undergoes cleavage because of the acidic environment (pH ~4) and releases the DNA-disrupting payload.

Preclinical studies of inotuzumab ozogamicin showed up to 39-fold more potent induction of *in vitro* tumor cell death compared to unconjugated calicheamicin in CD22^+^ B cell lymphoma cells [142] and significant *in vivo* tumor regression in lymphoma models as a single agent and in combination with rituximab, cyclophosphamide, vincristine, and prednisone (CVP), or CHOP [141, 142]. These results were transposed in clinical trials confirming a promising anti-tumor activity. The initial phase I study in patients with FL and DLBCL exhibited ORR of 39% (68% for FL; 15% for DLBCL) for the 79 enrolled patients [143], results thereafter confirmed by a phase 2 study in refractory indolent lymphomas (NCT00868608) with an ORR reaching 67% [144]. Its combination with rituximab has been tested in a phase I/II study demonstrating high tolerability and significant activity with ORR of 20% for refractory aggressive lymphomas, 87% for FL, and 74% DLBCL (NCT00299494) [145]. The phase 1 study combining the ADC with the mTOR inhibitor temsirolimus showed a 40% (7/18) of ORR, all in FL patients, but a too high toxicity, mainly thrombocytopenia [146]. Rituximab-inotuzumab ozogamicin regimen has been compared to rituximab-chemotherapy in R/R aggressive B cell lymphomas (NCT01232556), however, this phase 3 study was terminated for futility [147]. A new study comparing the efficacy and safety of inotuzumab ozogamicin in combination with rituximab, cyclophosphamide, vincristine, and prednisolone for the treatment of chemotherapy-naive patients with DLBCL, who are not candidates for anthracycline-based treatment, is currently recruiting (NCT01679119).

Another anti-CD22 ADC studied in lymphoma is pinatuzumab vedotin (pina, DCDT2980S, RG-7593), containing a cleavable maleimidocaproyl-Val-Cit-PABC linker attached to the micro-tubule inhibitor MMAE with a DAR of four (Table 2) [132]. Similar to brentuximab vedotin and polatuzumab vedotin, the linker is cleaved by cathepsin once the ADC has entered the tumor cell, thus activating the antimitotic mechanism of action. After demonstrating to have comparable activity with standard of care such as CHOP *in vitro* and *in vivo* lymphoma models [132], pinatuzumab vedotin was investigated in a phase I trial as a single agent and in combination with rituximab in R/R DLBCL, indolent lymphomas and CLL. Response rates were respectively 25% and 17% as single-agent and rituximab combination in DLBCL and 42% and 33% in indolent lymphomas [148]. Interestingly a phase 2 randomized study (ROMULUS, NCT01691898) compared combinations of rituximab-polatuzumab vedotin (R-pola) to rituximab-pinatuzumab vedotin (R-pina) in patients with DLBCL and FL. Both regimens achieved a similar ORR, however, R-pola was chosen for further clinical development in lymphoma due to longer durations of response and better toxicity [149].

Moxetumomab pasudotox is a recombinant immunotoxin composed of the Fv fragment of an anti-CD22 monoclonal Ab and a 38-kDa fragment of *Pseudomonas* exotoxin A (PE38). Unlike other ADCs, which use a linker, moxetumomab pasudotox is produced using recombinant DNA techniques (Table 2) [136]. PE is a bacterial toxin secreted in the culture medium as a single polypeptide chain of 613 amino acids and composed of three major functional domains: the N-terminal receptor binding domain (domain I) that consists of domains Ia (1–252 amino acids) and Ib (365–404 amino acids), a translocation domain (domain II) containing a furin site necessary to release domain III from the cell-binding domain I and a catalytic domain (domain III) responsible for the ADP-ribosylating activity that inactivates elongation factor 2 (EF-2). Four disulphide bridges are distributed among them, two located in domain Ia and the other for each domain Ib and II [150]. Upon binding to CD22, moxetumomab pasudotox is internalized through clathrin-coated pits into the endocytic compartment where the reduction of the disulfide bond in domain II and its subsequent furin-catalyzed cleavage release the domain III. The carboxyl terminal Lys residue at position 613 is then removed, creating a toxin fragment that inhibits protein synthesis by transferring the ADP-ribose moiety to EF-2, interrupting translation and leading to cell apoptosis [151].

Moxetumomab pasudotox is the more active form of the predecessor recombinant immunotoxin BL22 (also called CAT-3888), thanks to the substitution of serine-serine-tyrosine in antigen-binding site of the heavy chain with threonine-histidine-tryptophan. This resulted in a 14-fold increased binding affinity for CD22 and up to 50-fold improvement in cytotoxicity in HCL and CLL.

A single-arm phase 3 study in HCL (NCT01829711) evaluating moxetumomab pasudotox as monotherapy in heavily treated HCL patients (> 3 lines of therapy, including 100% with previous purine nucleoside analog use and 75% with prior rituximab exposure) met its primary endpoint achieving 30% durable CR rate, defined as maintenance of hematologic remission more than 180 days after assessment by independent review committees of CR and 75% ORR with a median follow-up of 16.7 months [152]. These results led to moxetumomab pasudotox FDA approval in September 2018 for adult patients with R/R HCL who received at least two prior systemic therapies, including treatment with a purine nucleoside analogue [153]. A phase I study is currently evaluating its combination with rituximab (NCT03805932). Other early phase trials that investigated the efficacy of moxetumomab pasudotox in hematologic malignancies were terminated early due to prioritization of resources (NCT01030536, NCT00587457, NCT00587015).

The cysteine-engineered version of the humanized anti-CD22 monoclonal Ab epratuzumab (hLL2) combined with the cathepsin B-cleavable Val-Ala PBD payload tesirine (SG3249) constitutes the promising ADC epratuzumab-cys-tesirine (Table 2). With a DAR of 1.74, epratuzumab-cys-tesirine shows potent and specific *in vitro* and *in vivo* activity against different CD22-expressing human lymphoma models (Ramos, Daudi, WSU-DLCL2, and SU-DHL-4), inducing DNA inter-strand cross-linking after a 2-h exposure period. In particular, a dose-dependent and durable anti-tumor activity was observed in Ramos model, in which CR was reached in all ten treated mice after a single dose of epratuzumab-cys-tesirine at 1 mg/kg. Epratuzumab-cys-tesirine is currently under evaluation in a phase I/II clinical trial in patients with R/R ALL (NCT03698552) [154].

### CD25

CD25, α-chain of the interleukin-2 (IL-2) receptor, is a type I transmembrane protein, expressed on activated and regulatory T cells, activated B cells, some thymocytes, myeloid precursors, and oligodendrocytes. Its function is critical in maintaining immune tolerance, in fact, decreased levels of CD25^+^ T cells in peripheral blood have been associated with systemic lupus erythematosus, psoriatic arthritis, and autoimmune liver diseases. On the other hand, CD25 is overexpressed in a variety of tumors, including lymphoma and leukemia, which explains the interest in it as a therapeutic target [64].

Camidanlumab tesirine is an ADC composed of a human IgG1 monoclonal Ab directed against human CD25 (HuMax-TAC), stochastically conjugated via the cathepsin-cleavable Val-Ala dipeptide linker to the PBD dimer warhead SG3199 with a DAR of 2.3 (Table 2) [155, 156]. Once internalized, the enzymatic release of the DNA cross-linker warhead payload led to cell death. Preclinical studies found high potency of camidanlumab tesirine both as a single agent and in combination in lymphoma cell lines. In particular, combined with the PI3K inhibitor copanlisib, the BCL2 inhibitor venetoclax, the HDAC inhibitor vorinostat, and the inhibitor of ribonucleotide reductase, gemcitabine resulted in synergistic activity [156, 157]. Furthermore, a single dose of camidanlumab tesirine resulted in dose-dependent and targeted antitumor activity in CD25-expressing lymphoma xenograft models and reached superior activity over brentuximab vedotin in xenografts of Karpas 299. Significant bystander killing of CD25-negative cells was also observed [155]. Based on these promising data in preclinical models, camidanlumab tesirine is being evaluated in several clinical trials. An initial multicenter, open-label, phase I study explored camidanlumab tesirine in patients with R/R HL and lymphoma, expressing CD25. Among 130 evaluable patients, the ORR was 58% with 29% reported CR, with respectively 71% and 42% of ORR and CR for HL patients, and 38% and 9% of ORR and CR for lymphoma patients (NCT02432235) [158]. A phase 2 trial in patients with R/R HL is currently ongoing (NCT04052997).

### CD37

CD37 is a transmembrane protein, a member of the tetraspanin superfamily, whose expression profile is restricted to lymphoid tissues, with the highest abundance in mature B cells and absence in early progenitor cells or terminally differentiated plasma cells [159]. Its role is crucial in regulating B cell survival, T cell/B cell interaction, IgG/IgA production and balance between immune responses and tolerance [160]. In addition, CD37 is highly expressed on the surface of malignant B cells, such as CLL and most lymphoma subtypes [159, 161].

Naratuximab emtansine is an ADC that incorporates a humanized IgG1 anti-CD37 monoclonal Ab conjugated to the maytansinoid DM1 via the thioether linker, *N*-succinimidyl-4-(*N*-maleimidomethyl) cyclohexane-1-carboxylate (SMCC), with proven preclinical anti-lymphoma activity (Table 2) [161, 162]. SMCC is a non-cleavable and membrane-permeable linker. It contains an amine-reactive *N*-hydroxysuccinimide (NHS ester) that forms an amide bond with the ε-amino group of Lys, a cyclohexane bridge that confers added stability and a maleimide group that reacts with the thiol group of the tubulin inhibitor maytansinoid DM1 to form thioether bonds. Naratuximab emtansine contains 3 molecules to 4 molecules of DM1 per Ab. Like the other member of maytansinoid family, the warhead DM1 induces G2/M cell cycle arrest after internalization, lysosomal processing, and Ab digestion which leads to the release of Lys-*N*-SMCC-DM1 as intracellular maytansinoid catabolite. Besides inducing potent *in vitro* B cell depletion, naratuximab emtansine showed comparable or better activity than the anti-CD20 Ab rituximab, the combination of CVP or bendamustine in subcutaneous B cell lymphoma tumor xenografts [161]. It is worth mentioning that *CD37* has also been presented as a tumor suppressor gene, possibly down-regulated in some DLBCL patients [163, 164] and loss of CD37 expression due to its gene homozygous loss has been reported in a DLBCL cell line with acquired resistance to naratuximab emtansine [165].

Furthermore, naratuximab emtansine was explored in a multicenter, open-label, phase I trial as the first CD37-targeting ADC in patients with R/R B cell lymphomas. Five (13%) of 39 evaluable patients achieved an ORR, four of which occurred in the subgroup of patients with DLBCL [166]. The manageable safety profile and evidence of activity support the development of this novel CD37-targeting agent in a phase 2 study in combination with rituximab which has completed recruitment with no posted results to our knowledge (NCT02564744).

### CD70

CD70 is a type II transmembrane glycoprotein, a member of the TNF ligand family, mainly expressed on activated T and B lymphocytes. Upon binding to CD27, CD70 promotes a positive costimulatory signaling mediated by the recruitment of TRAF proteins, which results in the proliferation, differentiation, and activation of T, B, and natural killer (NK) lymphocytes. A broad range of tumors, including 50–60% of lymphomas, overexpress CD70 [167, 168].

The extracellular domain of CD70 is the target of two ADCs based on humanized monoclonal Abs, vorsetuzumab mafodotin, and SGN-CD70A (Table 2). The first one consists of a non-cleavable maleimidocaproyl Phe linker and MMAF as payload. SGN-CD70A combines the anti-CD70 monoclonal Ab with the DNA-crosslinking PBD dimer drug, through a protease-cleavable linker. Preclinical investigation in T cell lymphomas showed that SGN-CD70A inhibited cell proliferation and induced high caspase activity in a dose-dependent manner [169]. Vorsetuzumab mafodotin was evaluated in a phase I dose-escalation study in patients with R/R CD70-positive lymphomas or metastatic renal cell carcinoma (RCC) without sufficient activity to support further clinical development (NCT01015911) [170]. Similarly, SGN-CD70A was studied in a phase I trial for the treatment of relapsed RCC, MCL, DLBCL, and FL of grade 3 (NCT02216890); due to modest activity and unfavorable safety profile, its applicability seemed limited [171].

### CD71

CD71 (transferring receptor protein 1) is a 95-kDa transmembrane glycoprotein, containing two disulfide-linked monomers joined by two disulfide bonds. Each monomer binds one transferrin molecule, creating an iron-Tf-TfR complex to facilitate iron uptake into cells by endocytosis. CD71 is highly expressed in almost all tumor types, including metastatic disease. However, since it is also homogeneously expressed on multiple normal cell types, including many progenitor hematological cells, CD71 is widely considered undruggable and the development of ADCs targeting CD71 could be challenging to develop [172]. CX-2029, a probody (PDC) drug conjugate targeting CD71, is a first-in-class drug candidate, containing the potent microtubule inhibitor MMAE (Table 2) [20, 172]. PDCs are ADC prodrugs, designed to remain inactive until activation by tumor-associated proteases in the neoplastic microenvironment [16, 20]. No lymphoma patient has been enrolled in the CX-2029 phase I study [20]; an on-going phase II trial (NCT03543813) is open for solid tumors or DLBCL [173].

### CD205

CD205 is a type I transmembrane glycoprotein, a member of the mannose receptor (MR) family. It is highly expressed on myeloid blood dendritic cells (DCs) and monocytes, but also on B and T cells, NK cells, DCs, and cortical epithelial cells. Immunohistochemistry (IHC) has also confirmed the increased expression of CD205 in different tumors, including lymphomas, leukemias, and multiple myeloma, compared with that in the corresponding normal tissues [174]. The first-in-class ADC MEN1309 is a humanized IgG1 Ab directed against CD205, conjugated through a cleavable *N*-succinimidyl-4-(2-pyridyldithio) butanoate linker to the maytansinoid microtubule disruptor drug DM4 (Table 2). A strong anti-tumor activity was observed in preclinical *in vitro* and *in vivo* models, with a median IC_50_ of 200 pmol/L in different lymphoma cell lines and tumor eradication in all mice with a single dose. MEN1309 also showed synergism when combined with other targeted agents such as rituximab [175]. Maximum tolerated dose has been reached, without progressing to cohort expansion for company decision (NCT03403725), however a second phase I study conducted in solid tumors is currently recruiting (NCT04064359).

### ROR1

ROR1 is a tyrosine-protein kinase transmembrane receptor, expressed on CLL, B cell lymphomas, acute leukemias, and many other tumors but not on most healthy adult tissues. It plays a functional role in promoting migration and tumor engraftment, motivating the development of ADCs with humanized monoclonal Abs specific for ROR1. Different ADCs are indeed in development (Table 2) [176–181]. Clinically, zilovertamab vedotin is, so far, the most advanced. It is a humanized IgG1 monoclonal Ab with a proteolytically cleavable linker, and the antimicrotubule MMAE [177–179, 182]. In the phase 1 study, 32 R/R lymphoma patients, unselected for ROR1 expression, were treated with zilovertamab vedotin [183]. The ORR was 47% in MCL (*n* = 15) and 60% in DLBCL (*n* = 5), while no clinical activity was seen in CLL (*n* = 7), FL (*n* = 3), and marginal zone lymphoma (*n* = 1) patients [183]. Among the on-going trials with zilovertamab vedotin, the ADC is being explored in combination with R-CHP in a phase 2 study for newly diagnosed DLBCL patients (NCT05406401), and in combination with standard of care for R/R DLBCL patients (NCT05139017).

### Multiple myeloma-related targets: BCMA, CD38, CD46, CD56, CD74, CD138

ADCs targeting BCMA, CD38, CD46, CD56, CD74, CD138 are not discussed here since this special issue contains another article devoted to ADCs in multiple myeloma [184], and these targets are mainly explored in this disease setting. However, it is important to highlight that the same targets are also expressed in subsets of lymphoma patients, and that the anti-BCMA belantamab mafodotin-blmf is one of the FDA approved ADCs.

## Conclusions

ADCs are revolutionary weapons in targeted therapy of malignancies, combining the selective targeting capability of monoclonal Abs with the high cytotoxicity of chemotherapeutics. Several key parameters must be considered during ADCs development, including appropriate target antigen, conjugation method efficacy, linker stability, drug potency as well as bystander effect, and on/off-target toxicities. Their growing relevance for the management of patients with solid tumors and hematological cancers is demonstrated by their fast growth rate in the market in the few last years. Lymphomas are an attractive target for the use of ADCs, bearing many surface markers that are largely restricted to the mature B and/or T cell lineage, as shown by the efficacy of anti-CD20 monoclonal Abs or CAR T cell therapy targeting CD19. So far, already three ADCs (the anti-CD30 brentuximab vedotin, the anti-CD79 polatuzumab vedotin, and the anti-CD19 loncastuximab tesirine) have been approved for lymphoma patients. An increasing number of ADCs with a multitude of antigen specificity, monoclonal Abs, linkers, and payloads are under preclinical or clinical evaluation in lymphomas, likely providing new active agents to treat patients. Since targets can be shared by ADCs, naked Abs, bispecific Abs, and CAR T cells [185–187], it will be important to define the optimal modality to offer each class of agents to the patients at the right time during their clinical course and to identify the populations that would most benefit in terms of anti-tumor activity, toxicity and financial costs.

## Abbreviations

Abs: antibodies
ABVD: doxorubicin, bleomycin, vinblastine, and dacarbazine
ADCs: antibody-drug conjugates
AEs: adverse events
Ala: alanine
ALCL: anaplastic large cell lymphoma
ALL: acute lymphocytic leukemia
auto-HSCT: autologous hematopoietic stem cell transplantation
BCMA: B cell maturation antigen
BR: bendamustine and rituximab
BV-AVD: brentuximab vedotin, doxorubicin, vinblastine, and dacarbazine
CAR: chimeric antigen receptor
CHOP: cyclophosphamide, doxorubicin, vincristine, and prednisone
Cit: citrulline
CLL: chronic lymphocytic leukemia
CR: complete response
CTCL: cutaneous T cell lymphoma
DAR: drug antibody ratio
DLBCL: diffuse large B cell lymphoma
DM1: maytansinoid, mertansine
DM4: maytansinoid, ravtansine
Fabs: antigen-binding fragments
FDA: Food and Drug Administration
FL: follicular lymphoma
GSH: glutathione
HCL: hairy cell leukemia
HL: Hodgkin’s lymphoma
HR: hazard ratio
IC_50_: half maximal inhibitory concentration
IgG: immunoglobulin G
IRR: infusion-related reaction
Lys: lysine
MCL: mantle cell lymphoma
MF: mycosis fungoides
MMAF: monomethyl auristatin F
ORRs: overall response rates
PABC: *p*-amino benzyloxycarbonyl
PBD: pyrrolobenzodiazepine
PFS: progression-free survival
Phe: phenylalanine
PI3K: phosphoinositide 3-kinase
PPA: norephedrine
PTCL: peripheral T cell lymphoma
R/R: relapsed or refractory
ROR1: receptor tyrosine kinase-like orphan receptor 1
SPDB: *N*-succinimidyl 4-(2-pyridyldithio)butyrate
Val: valine

## Declarations

### Copyright

© The Author(s) 2022.
